# Nine weeks of supplementation with a multi-nutrient product augments gains in lean mass, strength, and muscular performance in resistance trained men

**DOI:** 10.1186/1550-2783-7-40

**Published:** 2010-12-16

**Authors:** Stephen M Schmitz, Jennifer E Hofheins, Robert Lemieux

**Affiliations:** 1 Supplement Safety Solutions, 47 Wildwood Drive, Bedford, MA 01730, USA; 2 The Center for Applied Health Sciences, Division of Sports Nutrition and Exercise Science, 3624 West Market Street, Suite 104, Fairlawn, OH 44333, USA

## Abstract

**Background:**

The purpose of this study was to compare the effects of supplementation with Gaspari Nutrition's SOmaxP Maximum Performance™ (SOmaxP) versus a comparator product (CP) containing an equal amount of creatine (4 g), carbohydrate (39 g maltodextrin), and protein (7 g whey protein hydrolysate) on muscular strength, muscular endurance, and body composition during nine weeks of intense resistance training.

**Methods:**

Using a prospective, randomized, double-blind design, 20 healthy men (mean ± SD age, height, weight, % body fat: 22.9 ± 2.6 y, 178.4 ± 5.7 cm, 80.5 ± 6.6 kg, 16.6 ± 4.0%) were matched for age, body weight, resistance training history, bench press strength, bench press endurance, and percent body fat and then randomly assigned via the ABBA procedure to ingest 1/2 scoop (dissolved in 15 oz water) of SOmaxP or CP prior to, and another 1/2 scoop (dissolved in 15 oz water) during resistance exercise. Body composition (DEXA), muscular performance (1-RM bench press and repetitions to failure [RTF: 3 sets × baseline body weight, 60-sec rest between sets]), and clinical blood chemistries were measured at baseline and after nine weeks of supplementation and training. Subjects were required to maintain their normal dietary habits and follow a specific, progressive overload resistance training program (4-days/wk, upper body/lower body split) during the study. An intent-to-treat approach was used and data were analyzed via ANCOVA using baseline values as the covariate. Statistical significance was set *a priori *at p ≤ 0.05.

**Results:**

When adjusted for initial differences, significant between group post-test means were noted in: 1-RM bench press (SOmaxP: 133.3 ± 1.3 kg [19.8% increase] vs. CP: 128.5 ± 1.3 kg [15.3% increase]; p < 0.019); lean mass (SOmaxP: 64.1 ± 0.4 kg [2.4% increase] vs. 62.8 ± 0.4 kg [0.27% increase], p < 0.049); RTF (SOmaxP: 33.3 ± 1.1 reps [44.8% increase] vs. 27.8 ± 1.1 reps [20.9% increase], p < 0.004); and fat mass (SOmaxP: 12.06 ± 0.53 kg [9.8% decrease] vs. 13.90 ± 0.53 kg [4.1% increase], p < 0.024). No statistically significant differences in vital signs (heart rate, systolic and diastolic blood pressures) or clinical blood chemistries were noted.

**Conclusions:**

These data indicate that compared to CP, SOmaxP administration augments and increases gains in lean mass, bench press strength, and muscular performance during nine weeks of intense resistance training. Studies designed to confirm these results and clarify the molecular mechanisms by which SOmaxP exerts the observed salutary effects have begun. Both SOmaxP and the CP were well-tolerated, and no supplement safety issues were identified.

## Background

The use of pre- or peri-workout supplements among recreational and elite athletes have become increasingly popular due to studies suggesting improvements in aerobic and anaerobic performance and recommendations from expert panels in sports nutrition [1]. Among the most commonly used supplements for increasing muscular strength are those containing various creatine salts including creatine monohydrate [2], carbohydrate, protein [3], and amino acids [4], particularly branched chain amino acids (BCAA), for which evidence of effectiveness has been consistently seen in published studies [1]. Numerous studies have assessed the effectiveness of the individual supplements listed above, and have established a range of doses at which the specific supplement showed demonstrable effects. These studies have helped to establish minimal/threshold doses at which supplements exert their intended effects. Research data is most plentiful on supplementation with creatine monohydrate, carbohydrates, and protein and these three ingredients are consistently recommended by expert panels as ergogenic aids, and as such are the core constituent ingredients of many pre- and peri-workout supplements.

Based on the findings of such research and expert recommendations, supplement manufacturers have developed sports drinks combining the same three core ingredients and have added proprietary ingredients to be used in the peri-workout time period to increase muscle strength, lean mass, and/or endurance. Aside from the convenience of having multiple ingredients in one product, there is potential for the components to exert additive or synergistic effects. Because different dietary supplement products contain differing quantities of the core and proprietary components, it is often difficult to perform valid head-to-head studies. However, because most products purporting to build strength and/or endurance contain the same three core ingredients, and the preponderance of evidence suggests that these three ingredients are the most important contributors to observed ergogenic gains, then it is reasonable to assume that if similar quantities of the core ingredients were compared, a valid comparison could be made. If differences were found between two products, then a likely explanation for the difference would be some effect of the proprietary ingredients, since the core ingredients are matched by dose. Proprietary ingredients could contribute to a difference either by exerting independent effects or by enhancing the effects of the core ingredients in a differential way or both.

Size On Maximum Performance™ (SOmaxP) is a product manufactured by Gaspari Nutrition containing creatine, carbohydrate, whey protein and other proprietary ingredients, and was used during the peri-workout period only on the days when resistance training occurs. The comparator product was standardized to contain similar amounts of creatine, carbohydrate and whey protein. The study compared the effects of SOmaxP to a comparator product (CP), which was standardized to contain equal amounts of creatine (4 g creatine monohydrate), carbohydrate (39 g maltodextrin) and protein (7 g whey protein hydrolysate), and given with identical timing. We hypothesized that subjects in the SOmaxP groups would outperform the subjects in the CP during post-testing after adjusting for baseline differences.

## Methods

### Subjects

Twenty subjects, ten in each group, were randomized to receive either SOmaxP or CP during this 9-week study. Key elements of the inclusion criteria included: male or female subject in good health; aged between 18-45; a body fat of 10%-25% inclusive; who had undergone regular resistance training for at least two years; who had signed an informed consent; who were willing and able to comply with the training and supplement protocol; possessed normal vital signs; and had a fluent understanding of English. Physical activity levels and health history were determined using standardized questionnaires adapted from Kent State University, Purdue University, and Eastern Michigan University at baseline and weeks 3, 6 and 9. The protocol was in compliance with the Helsinki Declaration, and was approved by the IntegReview Ethical Review Board (Austin, TX). Although the inclusion criteria allowed for female subjects, no females enrolled in the study. The actual age range of subjects who participated in the study was 19-31 years.

Key exclusion criteria included: a history of various metabolic conditions or diseases; the concomitant use of a variety of medications, including but not limited to those with androgenic and/or anabolic effects; the use of nutritional supplements known to improve strength and/or muscle mass (e.g., creatine, HMB, androstenedione, DHEA, etc.) within six weeks prior to the start of the study; a weight gain or loss of more than 10 lbs. within the past 30 days; known allergy to any ingredients in SOmaxP Maximum Performance™ or CP; participation in other research studies within the last 30 days; the current use of tobacco products; and the presence of any orthopedic limitations or injuries.

### Study Design

The study was a prospective, randomized, double-blind, parallel-group clinical trial. Subjects were matched into two groups according to body mass, age, and resistance training experience. Subjects were then randomly assigned (via the ABBA procedure [5]) to receive either SOmaxP or CP. Following informed consent and prior to the first testing session, a research nutritionist and Certified Strength and Conditioning Specialist (CSCS) met with each subject and discussed in detail the strength training regimen, and nutritional and supplement requirements for the study period.

### Testing Sessions

Prior to pre-testing, subjects were instructed to refrain from heavy exercise for 48 hours and fast for at least 12-hours. The assessment of upper body muscular strength (1-RM) and repetitions to failure (RTF) testing was performed after a general warm-up of 3-5 minutes of light activity involving the muscle(s) to be tested (e.g., upper body ergometry prior to upper body strength testing). Next, the subject performed several minutes of static stretching exercises of the involved musculature. The subject then performed a specific warm-up set of 8 repetitions at approximately 50% of the perceived 1-RM followed by another set of 3 repetitions at 70% of the perceived 1-RM. Subsequent lifts were single repetitions of progressively heavier weights until failure. The initial increments in weight were evenly spaced and adjusted such that at least two single lift sets was performed between the three repetition warm-up set and the estimated 1-RM. At failure, a weight approximately midway between the last successful and failed lift was attempted. This process was repeated until the 1-RM was determined. The rest interval between sets was between 3-5 minutes (procedure modified from Brown et al., 2001) [6]. Results were obtained at baseline, and at week 3, 6 and 9. For testing at weeks 3, 6 and 9, in order to replicate pre-supplementation/baseline testing conditions as closely as possible, subjects were instructed to follow their previously recorded 3-day diet records, refrain from heavy exercise for 48 hours, and fast for at least 12-hours prior to the workout. Upper body muscle endurance was measured as the total repetitions completed during three successive sets of isotonic bench press at a load equal to 100% subjects' pre-testing body weight. Each set was separated by a one-minute rest period.

### Body Composition Assessment

Body composition was assessed at baseline, and weeks 3, 6 and 9. Standing height was determined using a wall-mounted stadiometer. Body weight was measured using a SECA™ Medical Scale. Lean mass and fat mass were assessed using dual energy x-ray absorptiometry (DEXA, General Electric LUNAR DPX Pro). For each subject, the same technician performed all four DEXA measurements.

### Supplementation Protocol

SOmaxP contains creatine monohydrate (4 g), carbohydrate (39 g), and whey protein (7 g), and a number of proprietary ingredients. Subjects randomized to the SOmaxP group took 1 serving of SOmaxP + 30 ounces of water starting 10-15 minutes before the workout and finishing before the end of the workout, and used the product only on the days when resistance training occurs. The comparator product (CP) was standardized to contain equal amounts of creatine monohydrate (4 g), carbohydrate (39 g maltodextrin) and protein (7 g whey protein), and given with 30 ounces of water, with identical timing, and similarly used only on resistance training days. The CP was virtually indistinguishable in taste, color and consistency to SOmaxP. The supplements were prepared in powder form and packaged in coded generic containers for double-blind administration by an independent company (Command Nutritionals, Fairfield, NJ). Compliance to the supplementation protocol was monitored by a research nurse/dietician who contacted the study subjects on a weekly basis by telephone. Subjects were required to bring in their supplement bottles on workout days at weeks 3, 6 and 9 for visual inspection by study personnel to assess compliance with the protocol.

### Side Effect Assessment

A questionnaire was completed at weeks 3, 6 and 9 (workout sessions 12, 24 and 36) to monitor individual changes in DOMS and assess potential adverse events and change in sleep habits, general attitude, irritability, appetite, thirst, muscle soreness, muscle cramping, stomach distress, and headache, as well as any other idiosyncratic responses to the supplementation/training protocol. If identified, events were recorded as adverse events. In addition, subjects were contacted on a weekly basis by phone contact to inquire if they had experienced any adverse events, and were told to call at any time during the study to report side effects.

### Dietary (Nutrition) Monitoring

The research dietitian met with each subject to explain the proper procedures for recording dietary intake. Each subject's baseline diet (3-days: two weekdays & one weekend day) was analyzed using the NutraBase IV Clinical Edition, (CyberSoft, Inc., Phoenix, AZ) to determine its energy and macronutrient content. Additional 3-day diet records were analyzed at weeks 3, 6 and 9 to verify that eating habits had remained consistent throughout the study.

### Resistance Training Protocol

**Figure 1:**
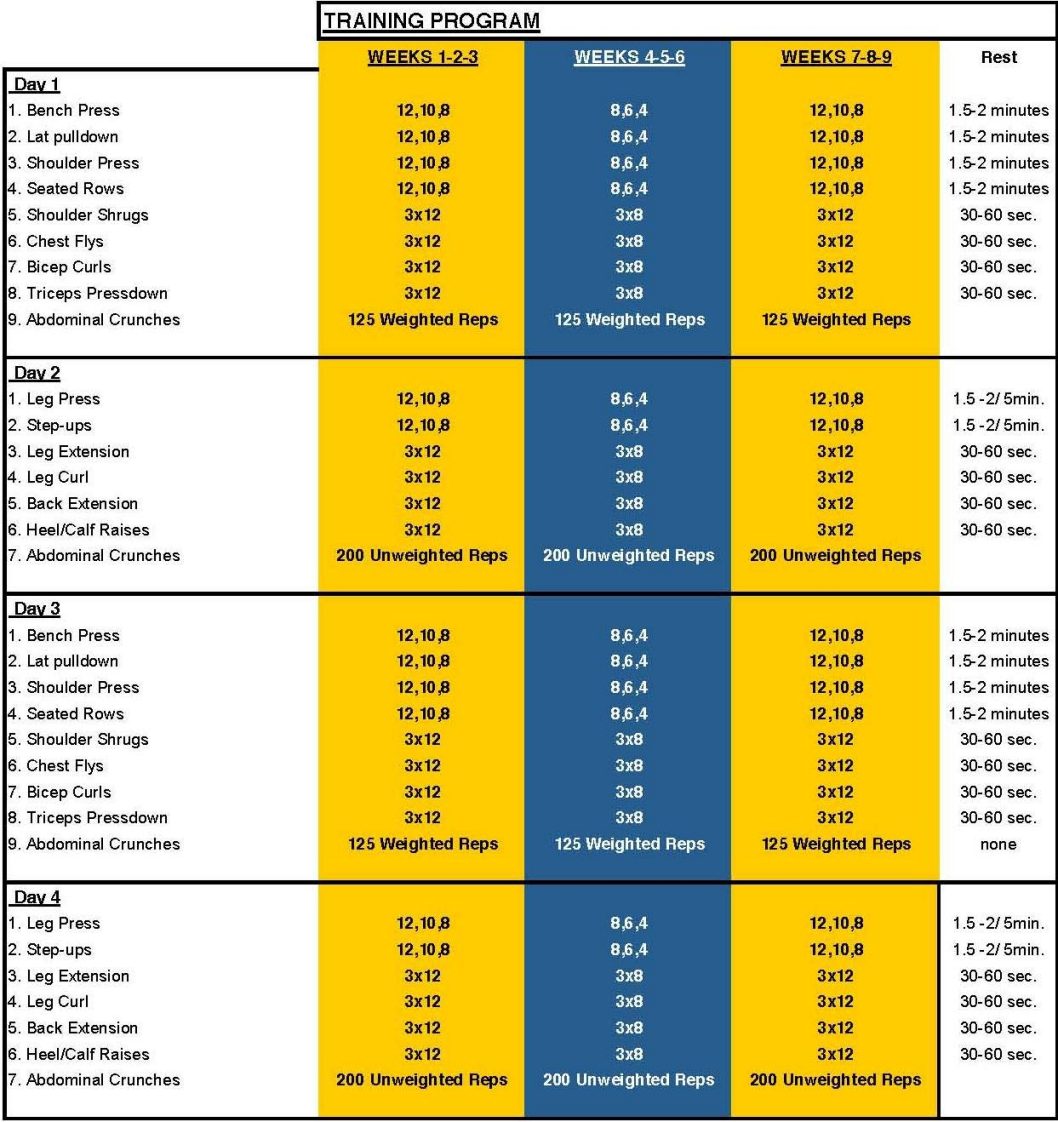
**Resistance Training Protocol**.

All subjects followed a specific 4-day per week workout designed by a Certified Strength and Conditioning Specialist (CSCS). The workout involved training the upper and lower body twice per week using a 4-day split (i.e., upper body^1^, lower body^1^, upper body^2^, lower body^2^) with gradual increases in volume and intensity. The workout consisted of at least 12 exercises, including but not limited to: bench press, lat pulldown, shoulder press, seated row, shoulder shrug, dip, biceps curl, triceps push down, leg press, squat, deadlift, lunge, leg curl, leg extension, and calf raise. For each exercise, subjects performed 3-6 sets of 8-15 repetitions with as much weight as they could handle with good form (typically 70-85% of the 1-repetition maximum). As subject strength and endurance improved, training resistances were progressively increased to maintain the required repetition range. Rest periods between exercises were 1-3 minutes, and between sets were 60-120 seconds. Training was conducted at the subject's local training facility, documented in training logs, and signed off by fitness instructors/gym personnel to verify compliance. Two different facilities were utilized and identical equipment was available at both facilities. In addition, at each session, the subject completed a physical activity question, which described their physical activity during the preceding month. A schematic of the training program is displayed below in Figure 1.

### Clinical Laboratory Chemical Analyses

Laboratory measures were performed at baseline, and weeks 3, 6 and 9. The tests included a complete blood count (CBC) with differential and platelet count, and a chemistry panel, which included sodium, potassium, chloride, carbon dioxide, calcium, AP, AST, ALT, bilirubin, glucose, blood urea nitrogen, creatinine, albumin, globulin, and estimated glomerular filtration rate, The lipid panel (total cholesterol, HDL- and LDL-cholesterol) was drawn at baseline and at week 9. Quest Diagnostics (Pittsburg, PA) was utilized to transport and analyze all blood samples.

### Statistical Analysis

Separate analyses of co-variance (ANCOVA), using baseline scores as the covariate were used to analyze between-group differences in body composition, muscular performance, and clinical markers of safety. Data was considered statistically significant when the probability of a type I error was less than or equal to 0.05 (P ≤ 0.05). If a significant group, treatment and/or interaction was observed, least significant differences (LSD) post-hoc analyses were performed to locate the pair-wise differences between means.

## Results

### Demographics

The demographic characteristics of the two cohorts were similar, and these are presented in Table 1. All 20 subjects were male, and the age range was 19-31 years. The mean values for age, height, weight, baseline fat percentage, blood pressure and resting heart rate were similar in the two cohorts.

**Table 1:** Baseline Demographic Characteristics

Parameter	SOmaxP	95% CI	Comparator (CP)	95% CI
Age (years)	21.9	20.5-23.3	23.9	21.9-25.9

Height (inches)	70.7	69.0-72.4	69.8	68.3-71.3

Weight (kg)	81.1	77.3-84.9	79.9	74.2-85.6

Fat percentage	16.78	14.0-19.6	16.45	13.4-19.5

Resting Heart Rate (bpm)	60.9	56.9-64.9	66.4	59.9-73.0

Blood pressure (mm Hg)	133/76	130-136/70-82	128/79	119-136/74-84

### Performance Measures

**Figure 2:**
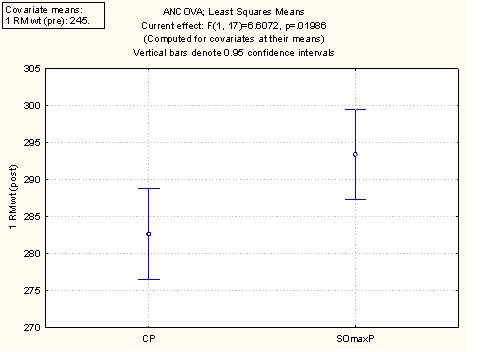
**ANCOVA for 1 Repetition Maximum Bench Press (1 RM)**.

**Figure 3:**
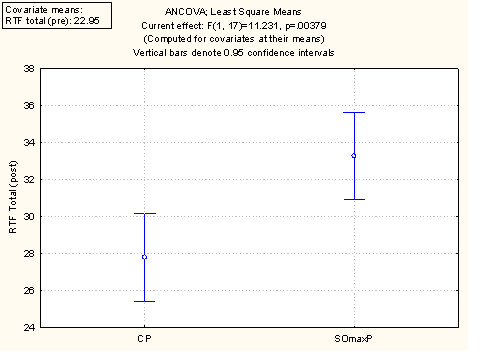
**ANCOVA for Repetitions to Failure (RTF)**.

**Figure 4:**
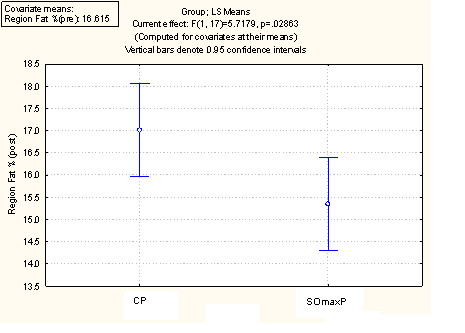
**ANCOVA for Percent Body Fat**.

**Figure 5:**
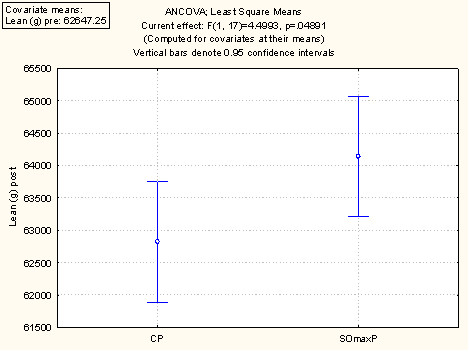
**ANCOVA for Lean Mass**.

**Figure 6:**
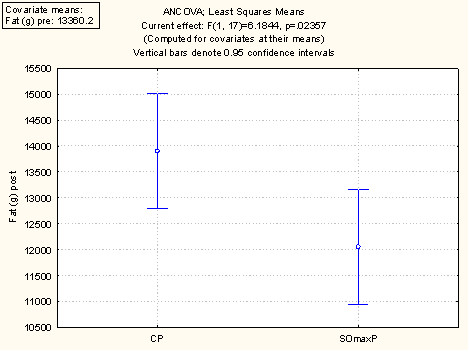
**ANCOVA for Fat Mass**.

A summary of the performance and outcome measures at baseline ("Pre") and at week 9 session ("Post") are presented in Table 2 and discussed below. The values are the mean values per cohort at baseline and week 9. Figure 2 displays these data using the least square mean ANCOVA analysis for 1 RM. Figure 3 displays the ANCOVA for Repititions to Failure (RTF). Figure 4 displays the ANCOVA for percent body fat. Figure 5 displays the ANCOVA for lean mass. Figure 6 displays the ANCOVA for fat mass. Statistically significant differences between the SOmaxP and CP cohorts were observed for 1 RM (p = 0.019), RTF (p = 0.004), body fat percent (p = 0.028), lean mass (p = 0.049), and fat mass (p = 0.023).

**Table 2:** Summary of Important Outcome Measures from Baseline to Week 9 (Workout session 36)

Measure	SOmaxP			CP			P-Value (ANCOVA)
	**Baseline**	**Week 9**	**%Change**	**Baseline**	**Week 9**	**%Change**	p-value (difference)*

**1-RM lbs (kg)**	233.5 (106.1)	283.5 (128.9)	21.4%	256.5 (116.6)	292.5 (132.9)	14.0%	0.019

RTF (total)**	19.6	30.25	54.3%	26.3	30.8	17.1%	0.004

**Body Fat %**	16.8	15.5	-7.7%	16.5	16.9	2.4%	0.028

**Lean Mass (kg)**	62.7	64.2	2.4%	62.6	62.8	0.3%	0.049

**Body Weight (kg)**	81.1	80.8	-0.2%	79.9	80.2	0.2%	0.22

**Fat Mass (kg)**	13.5	12.2	-9.6%	13.3	13.8	3.8%	0.023

*Via ANCOVA

**RTF (total) represents a sum of the 3 sets of bench press

The measures of muscular performance (1-RM and RTF total) increased in both the SOmaxP and CP cohorts, though by a higher percentage in the SOmaxP group. The 1 RM for the SOmaxP cohort increased from 233.5-283.5 lbs. [106.1-128.9 kg] from pre- to post-testing (21.4% increase), while the CP cohort increased from 256.5-292.5 lbs. [116.6-132.9 kg], (14.0% increase). The RTF for the SOmaxP cohort increased from 19.6 to 30.25 from pre- to post-testing (54.3% increase), while the CP cohort increased from 26.3 to 30.8 (17.1% increase).

**Figure 7:**
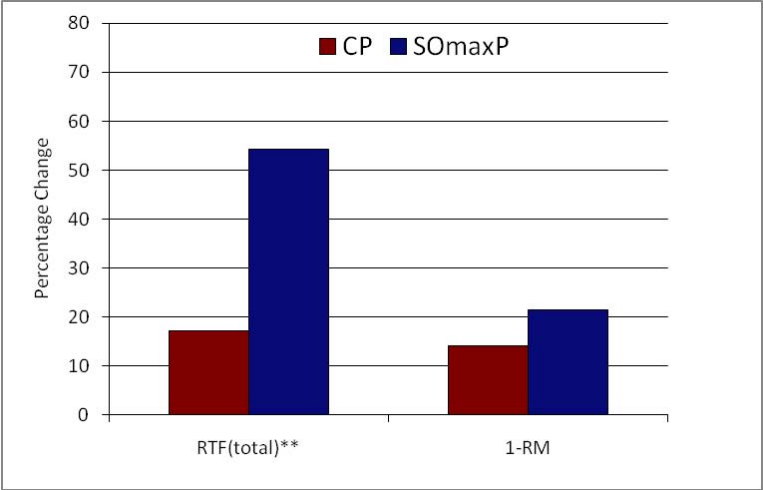
**Percentage Change from Baseline (Post minus Pre × 100) in Strength Measures**.

**Figure 8:**
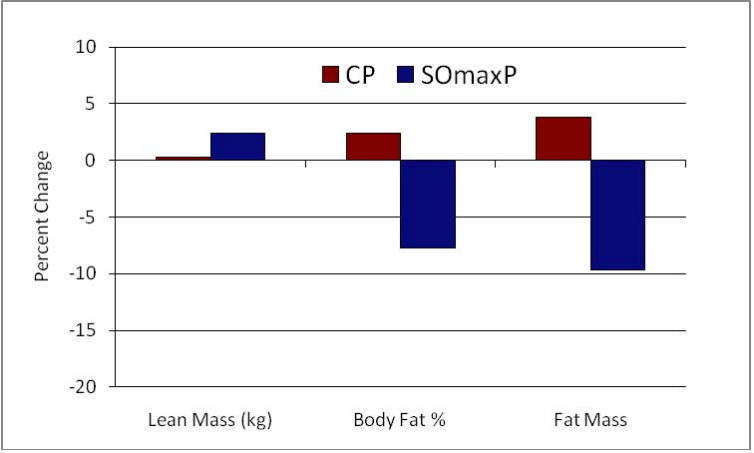
**Percentage Change from Baseline (Post minus Pre × 100) in Body Composition Measures**.

Several measures of body composition differed statistically between the two cohorts, with the SOmaxP cohorts demonstrating favorable improvements. The body fat percentage in the SOmaxP group decreased from 16.8% to 15.5% from pre- to post-testing (7.7% decrease), while the CP cohort increased slightly from 16.5% to 16.9% (2.4% increase). Lean body mass increased in the SOmaxP group from 62.7 kg to 64.2 kg (2.4% increase), while the CP cohort increased marginally from 62.6 kg to 62.8 kg (0.3% increase). Body weight did not change significantly in either group, with the SOmaxP group experiencing a drop of 1.5 kg from a baseline of 81.1 kg to 80.8 kg (0.2 kg decrease), while the CP cohort gained 1.5 kg from a baseline of 79.9 kg to 80.2 kg (0.2 kg increase). Finally, in the SOmaxP cohort, fat mass decreased from 13.5 kg to 12.2 kg (9.6% decrease), while the CP cohort increased from 13.3 kg to 13.8 kg (3.8% increase). The percentage change from baseline (Post minus Pre × 100) in strength measures (RTF(t) and 1-RM) are presented in Figure 7 below, and similar changes in body composition measures (lean mass, body fat percentage and fat mass) are presented in Figure 8.

There were no clinically meaningful changes in vital signs or laboratory results from baseline to Week 9. One subject experienced an adverse event. The subject was a 20 year-old male, (SOmaxP group) who experienced seasonal flu symptoms during Week 8 of the study. Symptoms included nausea, vomiting, and decreased appetite, and the events were not assessed as related to study product. Symptoms were resolved at the Week 9 post-testing visit. There were no significant changes in dietary intake for the subjects in either cohort, based on dietary diary evaluation.

## Discussion

This double-blind, comparator study showed that nine weeks of supplementation with SOmaxP resulted in statistically significant improvements in muscular performance (1-RM and RTF), decreases in body fat and fat mass, and increases in lean mass, versus a comparator product matched with similar amounts of creatine, carbohydrate and whey protein. Both the SOmaxP and CP were well-tolerated, and there were no changes in laboratory measures or vital signs during the study. There were no adverse events assessed as related to either product, and no significant changes in body weight occurred during the study period in either group.

The SOmaxP cohort experienced an increase in strength and a concomitant increase in lean muscle mass and loss in body fat, without a significant change in body weight. These changes are consistent with a desired anabolic effect. Improvements in strength were also noted with the CP, though significantly less than with SOmaxP. The dose of creatine in this study (4 g/workout or 16 g/week) for both the SOmaxP and CP cohorts is lower than what is recommended by some of the more commonly described creatine protocols^1^, and yet strength gains were noted in both the SOmaxP and CP groups. Typical protocols recommend ingesting approximately 0.3 g/kg/day of creatine monohydrate for 5-7 days as a loading dose (e.g., 5 g 4 times per day), followed by 3-5 g/day thereafter [7,8]. A few studies have found that a loading period was not necessary for increasing muscle creatine (3 g/day for 28 days) [9], or muscle size and strength (6 g/day for 12 weeks) [10,11]. A loading dose was not used in this study for either cohort. Data from the current study show measurable strength gains at a creatine dose of 16 g/week without a loading dose.

The CP cohort gained strength, but only had a slight increase in lean mass, body fat % and body weight. A possible explanation for this is that the CP group, taking a similar 16 g/week of creatine monohydrate experienced physiologic changes sufficient to increase strength, but not sufficient to measurably increase lean mass. This finding is consistent with work by Rawson et al. (2010), who found that subjects who received low dose creatine (2.3 g/day or 16.1 g/week) for six weeks, experienced a significant increase in plasma creatine, and statistically significant enhanced fatigue resistance without weight gain compared to a matched placebo group [12].

There are several possible explanations for the statistically significant difference between the SOmaxP group and CP, and these may be explained in part by several of the proprietary ingredients. SOmaxP contains a large quantity of branched chain amino acids. Branched chain amino acids (BCAAs), particularly leucine, have been shown to have anabolic effects, presumably through reducing protein breakdown [13]. BCAAs have also been shown to increase the lactate threshold during an incremental exercise test in trained individuals [14]. Blood lactate concentrations increase significantly during intense exercise as anaerobic glycolysis becomes the dominant energy pathway [15]. In addition, the combined ingestion of protein and leucine with carbohydrate has been shown to increase post exercise muscle protein in male subjects [16]. BCAAs also activate key enzymes in protein synthesis [17], and act in a synergistic fashion with insulin to allow skeletal muscle to coordinate protein synthesis [18].

In addition, SOmaxP contains isomaltulose (palatinose) as part of its carbohydrate moiety. This carbohydrate is present in honey and has been associated with delayed digestion and absorption, which may account for the difference in body fat changes between the SOmaxP group and the CP group. Oizumi and colleagues (2007) developed a palatinose-based balanced formula (PBF) for use in human subjects with impaired glucose tolerance [19]. During a 12-week cross-over study of dietary intervention in 23 subjects with impaired glucose tolerance, the authors found that A 250 kcal can of PBF once per day had beneficial effects on serum free fatty acid levels and visceral fat area. Visceral fat area decreased by 17.1% in the PBF period compared to 5.1% in the control period. Abdominal fat area decreased by 7.7% in the PBF interval while gaining 3.7% in the control period. Free fatty acids decreased by 22% in the PBF intervention, while increasing by 18.7% during the control period, and the 2-hour post-prandial glucose level decreased by 15.7% in the PBF intervention group while increasing by 0.8% in the control period. A possible mechanism for this finding was described in an animal study by Matsuo et al. (2007), who found that a palatinose-based liquid formula suppressed postprandial glucose level and reduced visceral fat accumulation compared to a standard formula [20]. These data suggest that palatinose-based carbohydrates may have beneficial effects on fatty acid and glucose metabolism.

In addition, Achten et al. (2007) compared the oxidation rates from orally ingested sucrose and palatinose (250 kcal) during moderately intense exercise [21]. The authors found that in trained athletes cycling for 150 minutes at approximately 60% of VO_2 _max experienced significantly lower oxygen consumption with palatinose compared to sucrose, resulting in a lower plasma insulin response at 30 minutes compared to sucrose. Subjects consumed either water or 1 of 2 carbohydrate solutions (sucrose or isomaltulose) providing 1.1 g/min of carbohydrate. The authors concluded that the lower carbohydrate delivery and a small difference in plasma insulin may have resulted in a higher endogenous carbohydrate use and higher fat oxidation during the isomaltulose trial than during the sucrose trial.

Another possible ingredient the SOmaxP that may contribute to the results of this study is L-ornithine-L-aspartate (LOLA), a substance shown to be effective in lowering blood ammonia concentration, particularly in patients with hepatic encephalopathy [22]. LOLA was administered at a dose of 20 g/day dissolved in 250 mL of 5% fructose solution and infused intravenously for a period of 4 hours during 7 consecutive days with a superimposed protein load at the end of the daily treatment period. Treatment was associated with a significant decrease in cerebral ammonia levels, which have been shown to be increased in subjects undergoing prolonged exercise [23]. Secher and colleagues (2008) reviewed the changes in cerebral blood flow and metabolism, and suggested that ammonia accumulation played a likely role in the development of what is known as central fatigue [24]. The efficacy of both oral and parenteral LOLA was confirmed by randomized, placebo-controlled, double-blind studies in patients with manifest hepatic encephalopathy and hyperammonemia [25]. The drug was able to reduce high blood ammonia levels induced either by ammonium chloride or protein ingestion or existing as a clinical complication of cirrhosis *per se*. Furthermore, LOLA improved performance in Number Connection Test-A as well as mental state gradation in patients with more advanced hepatic encephalopathy. Stauch et al (1998) found an improvement in cerebral ammonia levels compared to placebo using an oral dose of 6 gm per day [26].

In another published trial, LOLA decreased protein breakdown and stimulated protein synthesis in muscle in patients with hepatic encephalopathy [27]. The therapy had minimal side effects, increasing with higher intravenously administered dosages, and was well-tolerated after oral and parenteral administration. It is unclear if these results are generalizable to a healthy population, but the encephalopathy studies show that LOLA clearly has beneficial effects on the central nervous system and could possibly have an effect on central fatigue.

We acknowledge some limitations to the study. No females enrolled in the study, although some were approached for possible inclusion. The study group was small and homogenous, with a relatively tight age range, on the younger side of the eligibility criteria. No attempts were made to identify the physiologic mechanism for any differences between the two groups. The study attempted to control for the use of other supplements during the study, but did not perform any testing to verify non-use of other supplements.

## Conclusions

The use of SOmaxP four times per week for nine weeks resulted in statistically significant improvements in strength, muscle endurance, lean muscle mass, and percentage body fat versus a comparator with identical quantities of creatine, whey protein and carbohydrate. Given that the quantities of the core components were identical, and these components are presumed to contribute most to ergogenic effects, the differences between the SOmaxP and CP groups may be due to additive or synergistic effects of the proprietary ingredients in SOmaxP. Additional research is needed to further elucidate these effects. A double-blinded, comparator controlled study of six weeks duration which includes muscle biopsy measurements is currently underway to examine and possibly help identify genetic and pharmacological mechanisms by which SOmaxP may exert these effects.

## Affiliations

S. Schmitz is not affiliated with any institution. J. Hofheins and R. Lemieux are associated with The Center for Applied Health Sciences, Division of Sports Nutrition and Exercise Science. Mr. Lemieux works as the strength coach for Kent State University.

## Competing interests

Stephen Schmitz declares he has a potential competing interest as he is non-employee, part-time, paid consultant for Gaspari Nutrition, working specifically in the areas of dietary supplement adverse event monitoring and reporting for the company. Jennifer Hofheins and Robert Lemeiux declare that they are employed by the Center for Applied Health Sciences, which conducted the study. However, neither individual was compensated above and beyond their customary amount as a result of this study. Gaspari Nutrition is paying the JISSN article processing charges; however, no Gaspari Nutrition employee was involved in the writing of this article.

## Authors' contributions

SS was the primary author of the manuscript. JH worked at the study site, was involved in subject recruitment, data collection and editing of the manuscript. RL developed the workout routine for the protocol. All three authors have read and approved the manuscript.
